# Analecta Minora

**Published:** 1825-07-01

**Authors:** 


### XIII.

### ANALECTA MINORA,

OR

### *MINOR PERISCOPE.*

——— sparsa Colligens.

1. *Nitre in Menorrhagia.* At a late sitting of the "*Athenée de Medecine*," M. Goupil, M. Martinet, M. Deslandes, and others, related many cases where nitrate of potash in large doses, that is (from a scruple to a drachm or even more, three or four times a day) arrested menorrhagia after the usual means had been tried in vain. The last-mentioned physician reported his success with this medicine, in similar doses, when employed in cases of hæmoptysis. As nitre may be given with acids in large doses, in pulmonary hæmorrhages, without interrupting other means, as blood-letting, &c. it ought to be borne in mind by the practitioner, upon such occasions.

2. *Iodine in Sulphureous Springs.* M. Cantu, Professor of Chemistry in the University of Turin, has discovered that iodine, in the form of hydriodate, is contained in all sulphureous springs—at least, in all that he has analyzed, and he has examined a considerable number. This will account for the efficacy (in part at least) of these waters in glandular and visceral obstructions, and analogous diseases.

He comes to the following conclusions :—1mo. That iodine is most probably a constituent of all natural sulfureous waters containing the hydrochlorates. 2ndo. That the iodine is found, in these waters, in the state of hydriodate. 3tio. That the sulphuretted hydrogen, by a prolonged action on the hydrochlorates, and by other circumstances favourable to chemical reaction, may possibly give rise to the formation of iodine in these waters

4to. That iodine may, probably, not be a simple, but a compound body, one of whose elements may be chlorine. 5to. That the great efficacy of sulphureous waters in the cure of glandular and visceral obstructions, is owing in a great measure, to the presence of iodine in the form of hydriodate. 6to. That in this diluted state, and in combination with other substances contained in these waters, the iodine may act more efficaciously and less dangerously, than when in an insulated condition.

---

3. *Sangrado Practice.* The warm water system of the celebrated Sangrado has been lately revived in France, even to the letter. It was proposed by M. Cadet de Vaux, and put to the trial by M. Duchassin, a physician at Guise. It is for the cure of gout and rheumatism, and consists in the administration of 48 glasses of very hot water (de l'eau très chaude) taken in the course of 12 hours—that is, a glass every quarter of an hour. M. Duchassin avers, that he has been eye-witness of the cures performed in this way. Four cases are related in illustration, in the Gazette de Santé for May 5, 1825.

---

4. *Hydrophobia.** The following is one of the strongest instances, perhaps, on record, of the good effects of the actual cautery in preventing (it is very falsely styled *curing)* hydrophobia. It is related by Dr. L. Emiliani, in the work cited below.

Dom. Brizzi, a bleacher, had a large house-dog, who was kept chained in his court. This dog was bitten by another strange dog, who was wandering about. Thirty days after this event, the dog bit his master, his master's son, and a servant, and then shewed symptoms of hydrophobia, of which he died in a few days. The bitten persons took some remedies internally, which were said to be antidotes to hydrophobia, and six days elapsed before Dr. E. visited the family and explained to them the dreadful danger that probably awaited them. He proposed cauterising the eschars, to which operation Dom. Brizzi and his son assented, but the servant refused to comply, as he had been bitten just above the pubes. Professor Atti cauterised very profoundly the wounds of the two persons abovementioned, and kept up a considerable discharge from the parts by means of blistering ointment. These two escaped the disease; while the servant, on the other hand, was seized with hydrophobia, on the 27th day after the bite, and died, of course, in two or three days.

---

5. *Deafness in Old People.*† There came into the infirmary of the Salpetrière, an aged female of 82 years, who had been so extremely deaf as to be insensible to the noise of a trumpet at her ear. She died, and M. Pinel examined the organ of hearing with great care. The membrana tympani as compared with that of a person of the same age, but who had not been deaf, presented nothing particular, nor did the ossicula auditûs. On examining the labyrinth and the semicircular canals, no traces of a fluid could be found. This phenomenon led M. Pinel to look farther into the subject. On examining a number of persons whose hearing had been good before death, he invariably found the cochlea and semicircular canals filled with fluid. Some time afterwards, a female, who had been deaf for 30 years, particularly of the left ear, died, and was examined by our author. The membrana tympani appeared sound externally; but on opening it, the

---

* *Storia Medica di un Caso Raro, &c.* Transactions of a Society at Bologna, vol. iii.

† M. Pinel. Archives, Octobre, 1824.

cavity of the tympanum was found to contain purulent matter—the ossicula auditûs were partially carious—the membrane lining the cavity of the tympanum was red and inflamed—the cochlea and semicircular canals presented a similar state of inflammation, and the fenestra rotunda was eroded and open. Here it was evident that the deafness was occasioned by the chronic inflammation and its consequences, of the interior of the ear.

The same day, M. Pinel had an opportunity of examining the internal ear of a female, 84 years of age, who was partially deaf—or, in plainer terms, "hard of hearing." In this case, the only thing remarkable was, the paucity of fluid in the cochleæ and semicircular canals. In another person, who was deaf of the *right* ear, and who died scorbutic, our author found nothing particular in the interior of the *left* ear, while the lining membrane of the *defective ear* was discovered to be thickened, opake, and not a trace of fluid in the parts before mentioned.

Our author has, since that period, made many dissections of a similar kind, and with similar results. What may be the causes of this state of the internal ear he cannot take upon himself to say. Such are the facts which have presented themselves to his observation.

---

6. *Belladonna in Tic Douloureux.* Assistant Surgeon Henry, of the 66th, has related two cases of tic douloureux of the face, cured by the external application of belladonna to the nerve affected. Mr. Henry does not apprehend that this medicine has ever been used externally for the complaint before. But we can assure him that it has been tried hundreds of times. Mr. Bailey, of Harwich, published an octavo volume some years ago on "the use of belladonna in painful disorders of the head and face." Many practitioners, since that period, have tried it—sometimes with, but more frequently without success.

---

7. *Injecting Bodies with Whiskey.* Dr. Godman, of Philadelphia, has used common whiskey in injections, in preference to saline or acid solutions. This injection, he avers, answers all the purposes of the others, and is free from the objection of rusting the knives while dissecting the subject. —*Phil. Journ. No.* 18.

---

8. *Dissection Wounds.* Dr. Godman, in the same journal, informs us that the method pursued by the Philadelphia students, when they cut themselves, is, first to wash the wound well with warm water and soap—then to suck it forcibly for a considerable time, till the blood no longer flows—and lastly, to cover the wound with a piece of court-plaster till healed. After such procedure the dissector never entertains the least fear of bad consequences.

---

9. *Acupuncture.* A culpable piece of neglect in the Hotel Dieu, lately caused the death of a patient after acupuncture. A man, in Recamier's wards, had colica pictonum, for which the usual treatment was tried in vain. Three needles were, therefore, pushed into the abdomen—one at the epigastrium, and one on each side of the umbilicus. The needles were five inches long, half of which length penetrated the abdomen. The man did not remain quiet as he was ordered, but went to stool with the needles sticking in the abdomen. The consequence was that the two needles near the umbilicus, having no heads, escaped into the abdomen! The patient died the next day, and the reporters *say* there were no traces of inflammation in the abdomen. *Credat Judæus!—Revue Med. Avril.*

---

10. *Iron Nail swallowed.* A child, 18 months old, at Pembroke Dock, swallowed an iron nail about an inch long, having a rough head. Strong convulsions soon followed, and then spontaneous vomiting. These subsided, and on the fourth day, the stools became black and evidently ferruginous, with retention of urine. This last symptom being relieved, the child went on well till the 7th day, when vomiting and convulsions again recurred. The same train of symptoms took place once more on the fourteenth day, and, for the last time, on the 20th day; after which the child suffered no further inconvenience, and perfectly recovered its health. The black stools never appeared but once, and the nail could not be detected in the motions.—*Mr. Thomas. Med. and Phys. Journal.*

---

11. *Fractures of the Patella and Olecranon.* Mr. Amesbury has published some drawings of an apparatus for bringing into apposition, fractures of the abovementioned parts, in the May Number of the Medical Repository, to which we beg to point the attention of the surgical reader. We have seen the apparatus, and also an improvement which he has effected, since publication, in the straps corresponding to B in plate 1. and to D in plate 2.

---

